# Perceived teacher unfairness and students' school‐based adjustment: Exploring the mediating role of self‐handicapping and the moderating role of social support

**DOI:** 10.1111/bjep.70060

**Published:** 2026-01-16

**Authors:** Claudio Longobardi, Arooj Arshad, Matteo Angelo Fabris, Sofia Mastrokoukou

**Affiliations:** ^1^ Università Degli Studi di Torino Turin Italy; ^2^ Riphah Institute of Clinical Psychology Riphah International University Lahore Pakistan; ^3^ Universitas Mercatorum Rome Italy

**Keywords:** adolescents, psychological adjustment, self‐handicapping, social support, teacher unfairness

## Abstract

**Background:**

This study examined the pathways through which perceived teacher unfairness affects students' psychological adjustment, focusing on the mediating role of self‐handicapping and the moderating role of perceived social support.

**Aims:**

The study investigated whether self‐handicapping mediates the association between perceived teacher unfairness and students' psychological functioning, and whether perceived social support moderates this indirect relationship.

**Materials and Methods:**

The sample comprised 694 students aged 10–14 (M_age = 12.30, SD = 0.95; 52.2% female) attending public schools in Northern Italy. Participants completed validated self‐report measures assessing perceived teacher unfairness, self‐handicapping, perceived social support, and psychological functioning. Mediation and moderated mediation analyses were conducted.

**Results:**

Self‐handicapping significantly mediated the relationship between perceived teacher unfairness and psychological outcomes, including emotional symptoms, conduct problems, hyperactivity, and prosocial behaviour. Moderated mediation analyses indicated that perceived social support buffered the negative effects of self‐handicapping. Specifically, the indirect effects of teacher unfairness on emotional and behavioural difficulties via self‐handicapping were weaker among students reporting higher levels of social support, while no significant effects were found for peer relationship problems.

**Discussion:**

Teacher unfairness may contribute to maladjustment through increased self‐handicapping, while social support serves as a protective factor. Conclusion: Interventions promoting teacher–student fairness and strengthening support networks may enhance adolescent well‐being.

## INTRODUCTION

The teacher–student relationship is a well‐established protective factor for students' school adjustment during adolescence (Lin et al., 2025; Mastrokoukou et al., 2025). Not only can the teacher be an emotional reference point for students, providing support in the face of learning and developmental challenges (Fabris et al., 2022, 2023), but they also play a central role in shaping classroom dynamics and fostering student integration, often acting as an ‘invisible hand’ that influences the classroom climate (Krause & David Smith, 2023; Marengo et al., 2021). Foundational work by Wubbels and Brekelmans (2005, 2012) further demonstrated how teacher–student interpersonal styles shape classroom climate and students' adjustment, providing a historical anchor for contemporary research in this area.

In this regard, several studies indicate that a high‐quality teacher–student relationship, characterized by emotional closeness and low conflict, is typically associated with better psychological adjustment (Fabris et al., 2022, 2023) and higher academic performance (Emslander et al., 2025). In addition, through a positive relationship, the teacher can provide students with a relational model, which they may internalize and apply to peer relationships, thereby promoting respect, inclusion and a more positive classroom climate (Krause & David Smith, 2023; Marengo et al., 2021).

The teacher–student relationship can certainly deteriorate, and one construct that has emerged as increasingly relevant, yet under‐researched, is perceived teacher unfairness. Perceived teacher unfairness is defined as a student's subjective viewpoint of being treated with a lack of respect, integrity or equity in everyday teacher–student interactions (Lenzi et al., 2013; Gini et al., 2018). This construct encompasses relational unfairness, which relates to the quality of treatment between individuals rather than a structural inequity, such as grading or access to resources. Therefore, this study seeks to evaluate how the construct of perceived unfair treatment may negatively impact a student's psychological adjustment and motivate defensive forms of adjustment (i.e. self‐handicapping) while also considering social support as a protective factor.

### Teacher unfairness and psychological adjustment of adolescent students

In line with social‐cognitive models of stress applied to school contexts (Swearer & Hymel, 2015), several factors can contribute to the deterioration of adolescents' psychological adjustment in the school environment. Among them, perceived teacher unfairness remains under‐researched despite its potential impact. The term ‘teacher unfairness’ refers to students' subjective perception of being treated unfairly by their teachers (Gini et al., 2018). More specifically, relational unfairness pertains to the quality of interpersonal treatment; that is, whether students feel they are treated with fairness, honesty and respect (Lenzi et al., 2013). In this study, the construct is used to capture perceptions of unfair interpersonal treatment in daily teacher–student interactions, rather than broader structural inequities (e.g. biased grading or resource distribution). While unfairness may broadly encompass issues such as unequal rewards or preferential attention, the present study focuses specifically on students' subjective experiences of relational maltreatment, such as being treated harshly or disrespectfully.

This construct is particularly underexplored during adolescence, a limitation given the significant role teacher fairness may play in students' psychological well‐being. Fair treatment from teachers meets adolescents' core needs for acceptance and belonging (Cropanzano et al., 2001; Fabris et al., 2024) and fosters the development of self‐esteem (Cropanzano et al., 2001). A fair teacher–student relationship is also associated with greater academic engagement (Danielsen et al., 2010) and contributes to a more positive classroom climate (Murdock, 1999), thereby promoting psychological adjustment.

Conversely, the perception of being treated unfairly or differently by teachers may have profound negative consequences for adolescents' psychological and emotional functioning; especially in this developmental period marked by heightened sensitivity to social comparison (Gini et al., 2018). Negative relational experiences in school, such as teacher unfairness, can damage students' social connectedness and lead to feelings of isolation and alienation (Huang, 2020). According to Santinello et al. (2009), acts of relational unfairness can elicit stress‐related psychological responses, contributing to emotional and psychosomatic distress. Indeed, empirical evidence suggests that adolescents who perceive unfair treatment by teachers report higher emotional distress, increased anxiety (Huang, 2020) and more frequent psychosomatic symptoms (Gini et al., 2018; Mameli et al., 2018; Santinello et al., 2009). A recent longitudinal study found that adolescents perceiving high levels of teacher unfairness reported greater socio‐emotional difficulties and lower life satisfaction over time (Grew et al., 2024).

Furthermore, adolescents who perceive their teachers as unfair tend to experience more conflictual peer relationships and are more likely to engage in aggressive behaviours (Gini et al., 2024; Lenzi et al., 2014). This may be due to the development of a cynical and pessimistic view of authority figures, leading to the adoption of dominant and disrespectful interpersonal strategies, especially in conflict management.

Despite this growing evidence, there remains ambiguity regarding how ‘psychological adjustment’ should be understood in this context.

In the psychological literature, adjustment is most commonly defined in terms of internalizing and externalizing difficulties, such as emotional distress, anxiety, oppositional behaviour and hyperactivity (Compas et al., 2017). These dimensions capture adolescents' ability to regulate affect and behaviour in ways that are developmentally adaptive. At the same time, the school context provides a broader ecological frame in which relational dynamics also shape adjustment. For this reason, scholars increasingly emphasize that indices of positive functioning (e.g. prosocial behaviour) and relational risk (e.g. peer problems) represent important complementary outcomes when considering students' adaptation to the school environment (Goodman & Goodman, 2009; Oberle et al., 2011). Although these domains are not usually categorized under ‘psychological adjustment’, we believe that their inclusion, along with emotional and behavioural indicators, may provide a more ecologically valid and socially grounded account of adolescents' reactions to perceived teacher unfairness. In this study, we consider both core adjustment outcomes (emotional symptoms, conduct problems and hyperactivity) and complementary psychosocial indicators (prosocial behaviour and peer problems), and we treat psychological adjustment along with emotional and behavioural indicators as part of a broader investigation of school‐based adjustment.

Taken together, these strands of point to important but largely distinct areas of research. Perceived teacher unfairness has been linked to socio‐emotional difficulties, self‐deception has been widely studied as a defensively motivated academic strategy, and social support has consistently been seen as a protective buffer against stress. Yet there is surprisingly little evidence on how these domains intersect in adolescence, a developmental period in which adolescents are particularly sensitive to social judgment and relational context. The present study seeks to extend this body of work by examining these processes in a single moderated mediation framework. Specifically, we build on prior research by: (a) conceptualizing perceived teacher unfairness in relational rather than structural terms; (b) exploring self‐handicapping as a potential mediating process linking unfairness with adjustment outcomes; and (c) testing perceived social support as a moderator of these indirect effects, thereby identifying conditions under which unfairness may be less detrimental. This integrative framework represents a novel contribution, as it brings together constructs that have rarely been studied in concert. By adopting this integrative approach, the present study aims to provide a more comprehensive understanding of how teacher–student dynamics shape adolescents' psychological adjustment.

This preliminary study therefore investigates the mediating role of self‐handicapping in the relationship between perceived teacher unfairness and adolescents' psychological adjustment. The relevance of self‐handicapping, elaborated in the next section, stems from its potential to explain how perceived unfairness may affect academic self‐evaluation. Adolescents who feel unfairly treated may develop lower self‐confidence and experience aversive emotional states related to fears of failure and diminished control over academic tasks (Huang, 2020). In this context, self‐handicapping may emerge as a defensive strategy: a way to cope with anticipated failure by creating external explanations and thus protecting self‐esteem.

### Self‐handicapping: Exploring a possible mediating factor

Academic self‐handicapping is a maladaptive strategy individuals use to protect their self‐esteem from the possibility of academic failure (Berglas & Jones, 1978). To safeguard their self‐worth, individuals may engage in behaviours that hinder success, thereby creating plausible excuses to attribute failure to external rather than internal causes. Common self‐handicapping strategies include procrastination, effort withdrawal, substance use and setting unattainably high goals. These behaviours serve to create obstacles or ‘handicaps’ that can be cited as justifications in the event of failure.

According to Schwinger et al. (2022), self‐handicapping is rooted in students' uncertainty about their abilities and the associated threat to their self‐esteem. In practical terms, such behaviours allow students to deflect the causes of failure away from personal inadequacy, thus preserving a positive self‐image. Two distinct forms of self‐handicapping have been identified: behavioural and claimed (Leary & Shepperd, 1986). Behavioural self‐handicapping involves actively creating impediments to performance, such as substance use, studying in inappropriate environments or reducing study time. Claimed self‐handicapping, by contrast, involves verbal expressions of impediments, such as stating one is anxious, ill, shy or emotionally distressed, without necessarily engaging in behaviour that hinders performance (Leary & Shepperd, 1986; Wang et al., 2024).

Schwinger et al. (2022) argue that the experience of a threat to self‐esteem is a primary predictor of self‐handicapping. Two psychological factors that commonly elicit such threats are low expectations of academic success and concerns about social acceptance, particularly fear of negative evaluation by others. In line with self‐worth motivation theory (Covington, 1984), students often interpret academic outcomes as reflections of their intrinsic value and social worth. Therefore, when facing academic challenges, they may resort to self‐handicapping to buffer the psychological impact of potential failure.

From the perspective of achievement goal theory, Rhodewalt and Tragakis (2002) suggest that performance‐oriented goals (e.g. striving to appear competent or avoid looking incompetent) can heighten self‐esteem threats, as students become preoccupied with others' evaluations. By contrast, mastery‐oriented goals (i.e. the pursuit of personal growth and competence) shift attention from self‐image to task mastery, thereby reducing perceived threats to self‐esteem. Students high in failure avoidance or test anxiety may be especially prone to adopting performance‐oriented goals, thereby increasing the likelihood of self‐handicapping.

Overall, self‐handicapping appears to function as a self‐protective mechanism aimed at maintaining an image of competence and ensuring social acceptance. Within this framework, it is important to explore its relationship with perceived teacher unfairness, which has been shown to impair academic motivation and emotional well‐being. Students who perceive relational unfairness from teachers may feel unsupported, disengage from school and develop anxiety and fear of failure (Huang, 2020). These reactions are, in turn, associated with higher rates of self‐handicapping (Elliot & Church, 2003; Martin et al., 2015). Furthermore, perceived teacher unfairness can disrupt classroom social dynamics, increasing feelings of loneliness and social isolation (Huang, 2020). In this way, it may intensify the very psychological vulnerabilities, such as low self‐esteem and social insecurity, that predispose students to self‐handicapping.

Although self‐handicapping can temporarily preserve self‐esteem and intrinsic motivation (Wang et al., 2024), over time it is linked to adverse outcomes for adolescents' mental health and school adjustment. Studies have found that self‐handicapping is associated with increased internalizing symptoms, somatic complaints and substance use (Özçetin & Hiçdurmaz, 2016). Additionally, a cyclical relationship has been observed between self‐handicapping and low self‐esteem, compounding the risk of psychological distress (Zuckerman & Tsai, 2005). Finally, self‐handicapping is consistently linked with lower academic achievement (Schwinger et al., 2014), which may contribute to further distress and social withdrawal, ultimately undermining adolescents' overall well‐being.

### The moderating role of social support

While this study explores the potential association between perceived teacher unfairness and self‐handicapping, it is essential to consider the moderating role of social support. Social support refers to the interactive process through which individuals perceive themselves as valued, loved and integrated within a supportive social network. Scholars often emphasize perceived social support as a key indicator, reflecting individuals' overall assessment of the availability and quality of support within their networks.

Social support represents a critical psychological resource for adolescents, helping them to cope with external stressors and negative emotional experiences. Although peer relationships become increasingly salient during adolescence, support from parents, teachers and other significant adults remains vital (Badenes‐Ribera et al., 2019; Song et al., 2015). Given that perceived teacher unfairness can act as a stressor linked to poor psychological adjustment, the stress‐buffering model (Cohen & Wills, 1985) suggests that social support may mitigate its negative impact.

Empirical evidence supports this view. For example, high levels of perceived support from peers, parents and teachers have been shown to protect adolescents who have experienced abuse from declines in school adjustment (Cristescu & Băban, 2022). Similarly, greater perceived social support is associated with increased psychological well‐being (Tomás et al., 2020), fewer internalizing symptoms (Pössel et al., 2018), lower school burnout (Hoferichter et al., 2022) and reductions in hyperactivity and aggression (Demaray & Malecki, 2002). Longitudinal studies also demonstrate that social support positively influences school satisfaction (Jiang et al., 2013), academic performance (Ahmed et al., 2010), test anxiety and the adoption of performance‐avoidance goals (Song et al., 2015).

Although the moderating effect of social support has yet to be specifically tested in the relationship between teacher unfairness and self‐handicapping, some research suggests that social support buffers the impact of perceived injustice on maladaptive outcomes. For instance, in racial minority students, social support reduces the negative effects of perceived school injustice on aggressive behaviour (James et al., 2020).

Given that self‐handicapping is a strategy aimed at preserving self‐esteem, and considering that teacher unfairness negatively impacts students' well‐being and academic performance, it is plausible to hypothesize that perceived social support functions as a protective factor. Adolescents embedded in a supportive network may feel accepted even in the face of academic struggles, thus experiencing less pressure to use academic outcomes as indicators of self‐worth. As a result, they may be less inclined to engage in self‐handicapping behaviours and more likely to use adaptive coping strategies. Evidence further suggests that high levels of perceived social support are associated with improved social skills and academic outcomes (Ahmed et al., 2010; Demaray & Malecki, 2002). Moreover, social support can alter how individuals perceive and respond to stressors, enhancing their perceived capacity to meet demands and reducing the likelihood that a situation will be appraised as threatening.

## THE AIM OF THE STUDY

Given what has been outlined, the aim of our study is to investigate the possible association between perceived teacher unfairness and adolescents' psychological adjustment, testing the possible mediating role of self‐handicapping behaviour. Specifically, we expect that higher levels of perceived teacher unfairness will be associated with lower levels of adolescent psychological adjustment. Furthermore, we hypothesize that this relationship will be mediated by self‐handicapping behaviour. Specifically, we hypothesize that perceived teacher unfairness will be associated with an increase in self‐handicapping behaviour and that this, in turn, will be associated with a decrease in adolescents' psychological adjustment measures. Finally, the study aimed to test whether perceived social support moderates the indirect relationship between perceived teacher unfairness and psychological outcomes via self‐handicapping, thereby identifying social support as a potential protective factor in mitigating negative psychological effects.

### Hypotheses of the study

There is likely to be positive relationship between perceived teacher unfairness and psychological outcomes (prosocial behaviour).

There is likely to be negative relationship between perceived teacher unfairness and psychological outcomes (emotional symptoms, conduct problems, hyperactivity and peer relationship problems).

Self‐handicapping is likely to mediate between perceived teacher unfairness and psychological outcomes (prosocial behaviour, emotional symptoms, conduct problems, hyperactivity and peer relationship problems).

Social Support will likely moderate the indirect relationship between perceived teacher unfairness and psychological outcomes (prosocial behaviour, emotional symptoms, conduct problems, hyperactivity and peer relationship problems) via self‐handicapping.

## METHOD

### Sample

Initially, 750 students were approached using a purposive sampling strategy from various public educational institutions located in urban areas in northwestern Italy. Purposive sampling was chosen to deliberately select schools that could provide access to our target population of children and adolescents (ages 10–14) across the critical developmental period where teacher–student relationship dynamics significantly impact psychological outcomes. A total of 694 students completed the study questionnaires, resulting in a response rate of approximately 92.5%. The final sample included children and adolescents aged 10–14 years (M = 12.30, SD = .95), enrolled in grades 5 through 8. Of the participants, 52.2% were female (*N* = 362) and 47.8% were male (*N* = 332).

Participants were drawn from a diverse range of public schools, including both primary (scuola primaria, grades 1–5) and lower secondary institutions (scuola secondaria di primo grado, grades 6–8). In the Italian educational system, these schools are administered at the municipal level but operate within the broader framework of the regional school offices (Uffici Scolastici Regionali), which in turn fall under the governance of the Ministry of Education. This means that while day‐to‐day school management and student enrolment are overseen locally (typically by municipalities and school boards), curriculum standards, teacher recruitment and funding structures are nationally regulated. All schools included in this study were situated in municipalities within the northwestern Italian provinces selected for the research.

Based on self‐reported data, the sample represented a spectrum of middle‐ to lower‐income households, determined by parental education level and occupational status. All participants were native Italian speakers or fluent in the language of instruction and had no diagnosed cognitive or learning disabilities that would interfere with completing the survey instruments.

### Measures

#### The Perceived Teacher Unfairness Scale (PTUS; Lenzi et al., 2014)

Teachers' unfairness is assessed by using a 6‐item scale. Students are asked to rate how much they believed their teachers treated them fairly and respectfully (Lenzi et al., 2014). Although named ‘Perceived Teacher Unfairness’, the items of this scale primarily reflect students' perceptions of unfair interpersonal treatment in the classroom (e.g. being treated harshly or disrespectfully), rather than structural or distributive unfairness. The items were: ‘Teachers treat us fairly’ (reverse‐scored) and ‘Students are treated too harshly by teachers in our class’. Each participant answers on a 5‐point scale, with one denoting ‘completely disagree’ and five denoting ‘completely agree’. High scores reflect high perceptions of unfairness from teachers because positively keyed items are reverse‐scored. The Cronbach's alpha was *α* = .84. The current study's Cronbach alpha is *α* = .65.

#### The Multidimensional Scale of Perceived Social Support (MSPSS; Zimet et al., 1988)

People's level of support for their social circle is measured by MSPSS. It is a 12‐item scale with four items for each subscale, that is, family, friends and significant others. A seven‐point Likert scale, with one denoting ‘strongly disagree’ and seven denoting ‘strongly agree’, is used to record responses. Statements like ‘My family members help me’ and ‘My friends help me when I am having a hard time’ are among its contents. In this study, the composite score of the questionnaire was utilized. This scale's high internal consistency is indicated by Cronbach's alpha, which falls between *α* = .80 and *α* = .90. The Cronbach alpha of the current study is *α* = .89.

#### Self‐Handicapping Scale for Children (Waschbusch et al., 2007)

Children's tendency to use performance‐related excuses is measured by the Self‐Handicapping Scale for Children (SHSC) developed by Waschbusch et al. (2007). It is a 9‐item scale that presents various self‐handicapping scenarios, such as delaying tasks or not trying hard in order to justify poor performance. A four‐point Likert scale, with one denoting ‘totally disagree’ and four denoting ‘totally agree’, is used to record responses. Statements like ‘Some children put off doing their homework until the last minute so they can say that's why they didn't do as well as they had hoped’ are among its contents. In this study, a composite score of the questionnaire is utilized. This scale's acceptable internal consistency is indicated by Cronbach's alpha. The Cronbach alpha of the current study is *α* = .79.

#### The Strength and Difficulties Questionnaire (SDQ) (Goodman, 1997)

The Strength and Difficulties Questionnaire is developed by Goodman (1997) to assess behavioural, emotional and social relationships in children and adolescents. It consists of 25 items of which 20 items represent a total difficulty score having four subscales, including conduct problems, peer relationship problems, emotional symptoms and hyperactivity. The remaining five items represent prosocial behaviour. Parents/caregivers score each of the five items on each subscale as either never = 0, somewhat true = 1 or certainly true = 2. The analyses presented here are based on raw scores. In line with our conceptualization, emotional symptoms, conduct problems and hyperactivity were considered indicators of psychological adjustment, whereas prosocial behaviour and peer problems were analysed as complementary markers of social functioning.

The clinical cutoff raw scores (out of 10) for the subscales are as follows: Social Behaviour ≤4, Emotional Symptoms ≥4, Peer Problems ≥4, Conduct Problems ≥4 and Hyperactivity ≥7. The SDQ total score had good internal consistency (*α* = .75). The Cronbach alpha of the current study is *α* = .72.

#### Demographics data

The study used a demographic data sheet to gather basic information such as age, gender, education, grade and CGPA (Cumulative grade point average representing students' overall academic performance).

#### Ethical approval

The Ethical Committee for Scientific Research at the researchers' affiliated institution gave its approval to this study (IRB n. 0270661). The Helsinki Declaration's core principles (such as informed consent, data protection and confidentiality guarantees), the ethics standards necessary for human subjects' research and the rules set forth by the education management department were all adhered to. In addition, parental consent for the children's involvement in the study was acquired, and all participants and their parents were briefed on the study's guiding principles.

### Procedure

The research team, including the principal investigator and trained assistants, visited several elementary and lower secondary schools in northwestern Italy, to carry out data collection. These schools were selected through purposive sampling in collaboration with the school directors, who granted approval to conduct the study on their premises.

Students were asked to complete the questionnaires in paper format during regular class hours, within their own classrooms. Teachers were invited to complete their respective questionnaires during free periods or when they were available at school. All participants were informed that the questionnaire was anonymous and that their responses would be used solely for research purposes.

Participation in the study was entirely voluntary and uncompensated. Prior to data collection, written informed consent was obtained from parents or legal guardians, and student assent was also secured. Trained research staff provided clear instructions and remained present in classrooms to supervise the process and answer any questions. On average, it took participants approximately 10–15 min to complete the questionnaire.

At the conclusion of the session, the research team thanked the students, teachers and school directors for their cooperation and support in facilitating the study.

### Data analysis

Data analysis was performed using IBM SPSS Statistics version 23.0 (IBM Corp., Armonk, NY, USA). Descriptive statistics, including means and standard deviations, were calculated for each variable. Pearson's product–moment correlation coefficients were used to examine the associations among perceived teacher unfairness, social support, self‐handicapping and psychological outcomes. To test the hypothesized mediation and moderated mediation effects, the PROCESS macro for SPSS (Model 7) developed by Hayes (2018) was employed. This model allowed for testing the mediating role of self‐handicapping in the relationship between perceived teacher unfairness and psychological outcomes, as well as the moderating role of social support in the indirect path.

Separate models were run for each of the five subscales of psychological outcomes: prosocial behaviour, emotional symptoms, conduct problems, hyperactivity and peer relationship problems. Bootstrapping with 5000 samples was used to estimate the confidence intervals (CI) for the indirect and moderated mediation effects. Significant effects were determined based on 95% confidence intervals that did not include zero. The level of statistical significance was set at *p* < .05 for all analyses.

## RESULTS

### Descriptive statistics and correlations

To address H1 and H2, descriptive statistics and Pearson product–moment correlation analyses were conducted to examine the relationships between the main study variables. The results are presented in Table 1. Perceived teacher unfairness was found to be significantly positively associated with self‐handicapping and negative psychological outcomes, including emotional symptoms (*r* = .11, *p* < .05), conduct problems (*r* = .07, *p* < .05) and hyperactivity (*r* = .14, *p* < .05). By contrast, perceived teacher unfairness was negatively associated with social support (*r* = −.30, *p* < .01) and prosocial behaviour (*r* = −.09, *p* < .05).

**TABLE 1:** Descriptive statistics and correlations for study variable.

	M	*SD*	1	2	3	4	5	6	7	8
Perceived teacher unfairness	2.34	.79	–	−.30**	.16*	−.09*	.11*	.07*	.14*	.04
Social support	5.48	1.17		–	−.09*	.08*	−.12*	−.14*	−.17*	−.04
Self‐handicapping	2.82	.77			–	−.14*	.13*	.06	.12*	.03
Psychological outcomes										
Prosocial behaviour	1.52	1.90				–	.52**	.43**	.61**	.51**
Emotional symptoms	4.99	1.87					–	.58**	.46**	.64**
Conduct problems	4.67	2.03						–	.74**	.58**
Hyperactivity	5.32	1.94							–	.76**
Peer relationship problems	4.55	1.51								–

Abbreviations: M, mean; SD, standard deviation.

*p < .05. **p < .01. ***p < .001.

Regarding social support, significant negative correlations were observed with self‐handicapping (*r* = −.09, *p* < .05), emotional symptoms (*r* = −.12, *p* < .05), conduct problems (*r* = −.14, *p* < .05) and hyperactivity (*r* = −.17, *p* < .05). A significant positive association was found between social support and prosocial behaviour (*r* = .08, *p* < .05), indicating that higher levels of social support are associated with more adaptive psychological functioning.

Self‐handicapping was significantly negatively associated with prosocial behaviour (*r* = −.14, *p* < .05) and positively associated with emotional symptoms (*r* = .13, *p* < .05) and hyperactivity (*r* = .12, *p* < .05), suggesting that students who engage in self‐handicapping strategies tend to experience more internalizing and externalizing symptoms.

The correlation analysis revealed that perceived teacher unfairness was significantly positively related to self‐handicapping and negative psychological outcomes (emotional symptoms, conduct problems and hyperactivity), while it was negatively associated with social support and prosocial behaviour. In other words, higher levels of perceived teacher unfairness corresponded to greater self‐handicapping and maladjustment, alongside lower social support and prosocial tendencies.

In terms of social support, the findings revealed a significant negative relationship between self‐handicapping and negative psychological outcomes (emotional symptoms, conduct problems and hyperactivity); however, a significant positive relationship was found between prosocial behaviour. Indicating that more social support leads to increased prosocial behaviour. Furthermore, self‐handicapping is found to have a significant negative association with prosocial behaviour and a positive association with emotional symptoms and hyperactivity.

### Mediation analysis

To test H3, Model 4 of SPSS PROCESS Macro was used to perform mediation analysis. Five models were analysed, each incorporating the five subscales of psychological outcomes. The results are shown in Table 2.

**TABLE 2:** Path coefficients for the mediation model of self‐handicapping.

Paths	Direct effect		Indirect effect		95% CI	
	Unstandardized coefficient	SE	Unstandardized coefficient	SE	LL	UL
PTU→SH→PSB	−.16*	.09	−.04	.02	−.0822	−.013
PTU→SH→ES	.27*	.09	−.02*	.02	−.0501	−.005
PTU→SH→CP	.17*	.09	.02*	.01	.0035	.065
PTU→SH→HA	.28*	.08	.04*	.01	.0127	.084
PTU→SH→PP	.05	.07	.01	.01	−.0143	.031

Abbreviations: CI, confidence interval; CP, conduct problems; ES, emotional symptoms; HA, hyperactivity; LL, lower limit; PP, peer relationship problems; PSB, prosocial behaviour; PTU, perceived teacher unfairness; SH, self‐handicapping; UL, upper limit.

*p < .05; *p* < .001.

SPSS PROCESS Macro (Model 4) was used to analyse the mediation effect of self‐handicapping between perceived teacher unfairness and psychological outcomes using 5000 bootstrapped samples. The results revealed that self‐handicapping plays a significant mediating relationship with perceived teacher unfairness on psychological outcomes (prosocial behaviour, emotional symptoms, conduct problems, hyperactivity), while self‐handicapping did not play a mediating role with peer relationship problems.

### Moderated mediation analysis

**FIGURE 1:**
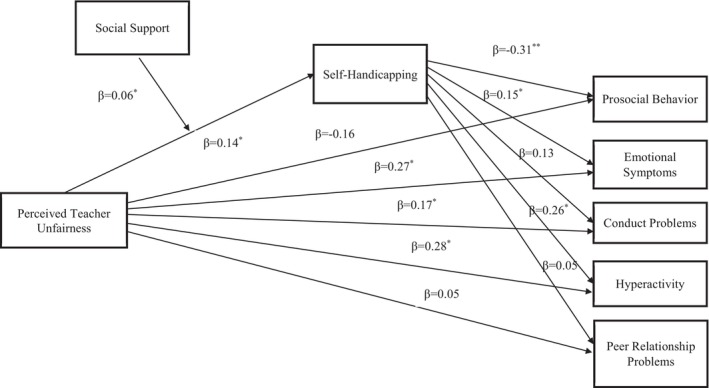
Moderated mediation model of perceived teacher unfairness and social support on psychological outcomes. The model includes perceived teacher unfairness and social support as exogenous variables and self‐handicapping, prosocial behaviour, emotional symptoms, conduct problems, hyperactivity and peer relationship problems as endogenous variables.

To examine H4, the moderated mediation analysis was run through SPSS PROCESS Macro with Model 7. Five models were run with five subscales of psychological outcomes. The model of moderated mediation analysis is shown in Figure 1, and the results are shown in Table 3.

**TABLE 3:** Path coefficients for the moderated mediation model of social support.

Probing moderated indirect relationships	Unstandardized coefficient	SE	95% CL	
			LL	UL
Index of moderated mediation (PSB)	−.03	.01	−.0482	−.001
Index of moderated mediation (ES)	−.00	.01	−.0202	−.001
Index of moderated mediation (CP)	.01	.00	.0021	.035
Index of moderated mediation (HA)	.02	.01	.0056	.048
Index of moderated mediation (PP)	.00	.01	−.0065	.018

*Note*: *p* < .05; *p* < .001.

Abbreviations: CI, confidence interval; CP, conduct problems; ES, emotional symptoms; HA, hyperactivity; LL, lower limit; PP, peer relationship problems; PSB, prosocial behaviour; UL, upper limit.

The analysis of moderated mediation was done by means of SPSS through PROCESS Macro (Model 7) with 5000 bootstrapped samples to test the conditional indirect effects, that is, it was tested to what extent social support moderates its indirect effect via the mediator (self‐handicapping) on psychological outcomes (prosocial behaviour, emotional symptoms, conduct problems, hyperactivity and peer relationship problems). This moderated mediation effect requires an interaction between social support and mediator self‐handicapping to be significant.

Moreover, the moderated mediation analysis explored significant interactions between self‐handicapping and social support, indicating that the negative effects of perceived teacher unfairness on psychological outcomes were buffered by social support. Specifically, prosocial behaviour, emotional symptoms, conduct problems and hyperactivity all showed significant interactions with social support and self‐handicapping, suggesting that social support mitigated the detrimental impact of self‐handicapping on these outcomes, whereas no significant effect was observed for peer relationship problems.

**FIGURE 2:**
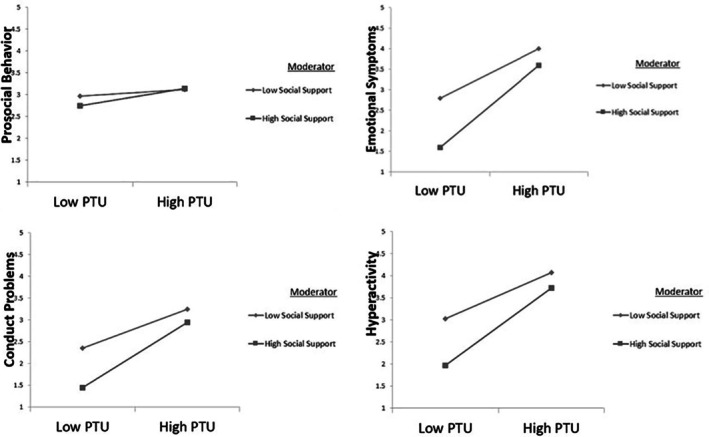
Graphical representation of the moderated mediation effects. The figure illustrates that students with higher perceived social support show fewer emotional symptoms, conduct problems and hyperactivity, even under high perceived teacher unfairness.

Figure 2 illustrates that students who have a high degree of social support show fewer emotional symptoms, conduct problems and hyperactivity, and a reduced reaction to perceived unfairness from teachers. By contrast, students with strong social support are more likely to act in a prosocial manner even when they perceive teacher unfairness. This implies that social support mitigates the detrimental effects of perceived teacher unfairness on psychological outcomes by acting as a protective factor.

In summary, the results indicated that perceived teacher unfairness was positively associated with psychological outcomes (emotional symptoms, conduct problems and hyperactivity) and negatively associated with prosocial behaviour. Self‐handicapping significantly mediated these relationships, supporting the hypothesized indirect effects. Moderation analysis showed that social support interacted with self‐handicapping to influence these outcomes, highlighting its role as a complex protective factor. Overall, these findings align with the initial hypotheses, confirming that perceived teacher unfairness adversely affects students' psychological outcomes, with self‐handicapping acting as a key mediator and social support serving as a moderator.

## DISCUSSION

The aim of our study was to investigate the association between perceived teacher unfairness and psychological adjustment in adolescent students, by examining the potential mediating role of self‐handicapping. Additionally, we explored whether this mediation is moderated by the perceived level of social support. This theoretical model had not previously been examined in the literature and thus constitutes the principal contribution of our exploratory work.

Our data support the first hypothesis, namely that perceived teacher unfairness is associated with lower psychological adjustment in the school context. More specifically, higher levels of perceived teacher unfairness were associated with increased emotional symptoms, conduct problems and hyperactivity, while also correlating negatively with prosocial behaviour. These results align with previous studies indicating that teacher unfairness can act as a psychological stressor with adverse effects on students' adjustment (Civitillo et al., 2024; Grew et al., 2024). The observed association between perceived teacher unfairness and emotional symptoms is consistent with prior findings linking it to anxiety (Huang, 2020), somatic symptoms (Gini et al., 2018; Mameli et al., 2018) and broader socio‐emotional difficulties in adolescents (Grew et al., 2024). It is plausible that when students perceive an unfair relationship with a teacher, they feel academically and emotionally unsupported, thereby increasing their psychological distress. Moreover, negative teacher–student interactions may be visible to peers, and being treated unfairly could increase students' social rejection or isolation (Wu et al., 2025).

The negative association between perceived teacher unfairness and prosocial behaviour, along with its positive association with hyperactivity and conduct problems, also aligns with literature suggesting that students perceiving high unfairness are more prone to aggressive and oppositional behaviours (Gini et al., 2024; Lenzi et al., 2014). It is possible that perceived unfairness contributes to a more negative view of authority and generates negative emotions, potentially manifesting as aggressive or defiant conduct. According to equity theory, adolescents who perceive unfair treatment may develop feelings of hostility and frustration, which can result in aggressive responses, particularly directed at the perceived source of injustice (Chory‐Assad & Paulsel, 2004; Vieno et al., 2011). In support of this, Lenzi et al. (2014) found that adolescents perceiving teacher unfairness were more likely to engage in bullying behaviours, possibly as a compensatory mechanism to restore perceived equity within the peer group.

While previous research supports a link between teacher unfairness and poor psychological adjustment, the mechanisms underlying this association have rarely been addressed. Our study provides novel insights into the mediating role of self‐handicapping in the relationship between perceived teacher unfairness and students' adjustment. To our knowledge, this is the first study to identify a significant positive association between teacher unfairness and self‐handicapping behaviours. Theoretical perspectives concur that self‐handicapping is a strategy used to protect self‐esteem from potential damage following academic failure. Previous research has shown that perceived unfairness in adolescence is generally associated with reduced self‐esteem (Zhao & Ngai, 2022). More specifically, unfair teacher behaviour may undermine students' perception of academic support, lowering engagement and increasing the risk of academic failure (Civitillo et al., 2024; Huang, 2020). Given that adolescents often perceive academic success as tied to self‐worth and peer acceptance, self‐handicapping may serve as a psychological defence strategy, allowing students to externalize failure and preserve a positive self‐concept.

Our findings support not only a direct association between perceived teacher unfairness and self‐handicapping, but also a mediating role of self‐handicapping in the relationship between teacher unfairness and multiple adjustment indicators. Specifically, self‐handicapping mediated the associations between teacher unfairness and lower prosocial behaviour, as well as higher emotional symptoms, conduct problems and hyperactivity. These results are consistent with previous studies linking self‐handicapping to anxiety (Greaven et al., 2000), psychosomatic complaints and substance use. Longitudinally, self‐handicapping is associated with increased negative affect and decreased self‐esteem (Zuckerman & Tsai, 2005), potentially initiating a self‐reinforcing cycle of psychological distress. Although self‐handicapping may initially protect self‐concept, the behaviours associated with it (e.g. procrastination, avoidance, disengagement) are likely to impair academic performance and increase emotional maladjustment (Schwinger et al., 2014). Moreover, self‐handicapping strategies such as withdrawal or substance use can hinder social functioning, contributing to further distress. Individuals with high levels of self‐handicapping often report elevated anxiety and somatic complaints, which may not only exacerbate distress but also diminish perceived social support and peer connectedness, leading to increased feelings of isolation.

We also examined whether perceived social support moderates the indirect effect of teacher unfairness on psychological adjustment via self‐handicapping. The underlying theoretical premise is that social support may buffer the stress induced by perceived unfairness and thereby mitigate its impact. Our results suggest that the association between teacher unfairness and psychological adjustment through self‐handicapping is stronger among adolescents with lower levels of perceived social support. By contrast, higher levels of perceived social support may provide access to resources that help adolescents cope with unfair treatment. For instance, students who perceive unfair teacher behaviour might still fulfil their relational and emotional needs through other meaningful relationships, such as with peers or family members, thereby buffering the impact of unfairness on school adjustment. Adolescents who feel socially supported tend to experience less loneliness, greater peer acceptance (Vargas‐Madriz & Konishi, 2021) and higher self‐esteem (Zhao et al., 2021). These factors may enhance resilience and reduce the need for defensive strategies like self‐handicapping. Additionally, previous research has linked perceived social support to better academic outcomes (Ahmed et al., 2010), lower test anxiety and weaker performance‐avoidance goals (Song et al., 2015). Consequently, students embedded in strong support networks may feel more motivated and better able to manage academic stressors, including unfair treatment by teachers.

Although this is the first study to examine the moderating role of social support in this specific model, our findings align with broader literature on perceived discrimination. For example, James et al. (2020) found that among racial minority students, social support mitigated the negative effects of perceived school injustice on aggressive behaviour.

In conclusion, our study offers initial empirical evidence supporting the mediating role of self‐handicapping in the relationship between perceived teacher unfairness and psychological adjustment, while also highlighting the moderating role of perceived social support. The findings suggest that adolescents may engage in self‐handicapping when they perceive a negative or unfair relationship with a teacher, particularly when social support is low. Such perceptions may reduce motivation and emotional support, acting as a barrier to academic success. Concern about academic performance under perceived threat may trigger self‐handicapping as a means of preserving self‐worth. Although these behaviours may be initially adaptive, over time they may impair school adjustment and contribute to emotional and behavioural difficulties.

### Limitations

Despite offering novel insights into the relationship between perceived teacher unfairness and psychological adjustment in adolescents, our study has several limitations that must be considered when interpreting the findings.

First, we employed a convenience sample of adolescents, which is not representative of the broader population of Italian adolescents. Future research should consider replicating this study with a representative sample or conducting cross‐cultural investigations to examine whether the observed associations hold across different cultural contexts.

Second, our cross‐sectional design prevents us from making causal inferences about the directionality of the relationships between variables. Longitudinal studies are therefore needed to explore how perceived teacher unfairness, self‐handicapping and psychological adjustment evolve over time and influence one another.

Third, the exclusive use of self‐report instruments may introduce biases related to social desirability, subjective interpretation of items and individual emotional states at the time of data collection. Incorporating additional data sources, such as teacher reports, peer evaluations or observational methods, would help validate and enrich our findings.

Additionally, self‐report questionnaires provide only quantitative data, limiting insight into students' subjective experiences. Future research could integrate qualitative methods (e.g. interviews or open‐ended surveys) to capture the nuanced ways adolescents perceive and internalize teacher unfairness and its consequences.

Finally, it is worth noting that our measures did not include contextual school‐level variables such as school climate, teacher–student ratio or teacher training on relational equity, factors that could potentially moderate the observed associations. Including such variables in future studies could provide a more comprehensive understanding of the school environment's role in adolescent psychological adjustment.

### Practical implications

Although further research is needed to confirm and expand upon our findings, several practical implications can be drawn for educational practice.

One of the clearest implications is the sensitisation of school staff, particularly teachers, to the potential psychological consequences of perceived unfairness for adolescent students; increased awareness may promote sensitivity to students' perceptions of fairness and facilitate more equitable and supportive relationships that could enhance students' psychological adjustment, when interacting with them.

In pedagogical contexts, professional development must go beyond knowledge about equity but also provide safe, inclusive opportunities to reflect, receive feedback and practice these interactions to build relationships in a respectful and inclusive manner. Professional learning opportunities for teachers may include structured modules on fairness in relationships, emotional self‐regulation (for self and students) in teaching and learning, and conflict resolution. These forms of professional learning aim to raise awareness of unfairness in practice and reduce contentious behaviours while developing a priority context that is inclusive and psychologically safe for learners.

Equally important, educators and school psychologists could more actively evaluate perceptions of student–teacher relationships when assessing risk factors for psychological maladjustment, including self‐harming behaviours. Consideration of these relational contexts could give meaning to intervention strategies that rely on relationships in combination with self‐control strategies that may indicate maladjustment. Furthermore, educators and psychologists might consider self‐injurious behaviour as an early indicator of emotional disturbance in certain learners who appear to report negative relational experiences with their teachers. Preventive approaches aimed at developing adaptive coping strategies and promoting self‐efficacy and resilience may include psychosocial or group counselling focused on strengthening and promoting learning orientations.

Finally, school‐wide interventions that provide a sense of belonging and foster a world of social support to mitigate any impact of teacher unfairness on perceived unfairness could be a helpful approach to minimizing the potential negative effects of perceived unfairness in teacher–student relationships, such as peer mentoring, engagement in extracurricular activities or family engagement as a proactive means of engagement for learners. In this context, fostering a supportive and fair school climate could serve as both a preventative and protective strategy for maintaining positive psychological adjustment in adolescents.

## CONCLUSION

Our study offers new insights into the mechanisms linking perceived teacher unfairness to adolescents' psychological adjustment. Specifically, the findings suggest that when students perceive unfair treatment by teachers, they may be more likely to engage in self‐handicapping behaviours, which in turn are associated with increased emotional and behavioural difficulties. Importantly, our results also indicate that perceived social support can moderate this association, acting as a protective factor that reduces the negative impact of perceived unfairness on students' adjustment.

These findings underscore the importance of fostering fair and supportive teacher–student relationships within school environments. Interventions aimed at enhancing teacher awareness of relational dynamics, promoting fairness in classroom management and strengthening students' social support networks could be beneficial. Ultimately, promoting relational equity and emotional support in educational settings may help mitigate psychological risks and support adolescents in achieving both academic and personal well‐being.

## AUTHOR CONTRIBUTIONS

**Claudio Longobardi:** Conceptualization; investigation; writing – review and editing; project administration; supervision. **Arooj Arshad:** Methodology; software; formal analysis. **Matteo Angelo Fabris:** Conceptualization; writing – original draft; data curation. **Sofia Mastrokoukou:** Investigation; writing – original draft; methodology; supervision.

## FUNDING INFORMATION

This study wasn't funded.

## CONFLICT OF INTEREST STATEMENT

The authors declare that they have no known competing financial interests or personal relationships that could have appeared to influence the work reported in this study.

## ETHICS STATEMENT

All procedures performed in studies involving human participants were in accordance with the ethical standards of the institutional and/or national research committee and with the 1964 Helsinki declaration and its later amendments or comparable ethical standards.

## INFORMED CONSENT

Informed consent was obtained from all individual participants included in the study.

## Data Availability

The data that support the findings of this study are available on request from the corresponding author. The data are not publicly available due to privacy or ethical restrictions.
